# Freezing Stallion Semen—What Do We Need to Focus on for the Future?

**DOI:** 10.3390/vetsci11020065

**Published:** 2024-02-02

**Authors:** Ziyad Al-Kass, Jane M. Morrell

**Affiliations:** 1 Clinical Sciences, Swedish University of Agricultural Sciences, Box 7054, SE-75007 Uppsala, Sweden; ziyad.al.kass@slu.se; 2 Department of Surgery and Theriogenology, College of Veterinary Medicine, University of Mosul, Mosul 41002, Iraq

**Keywords:** stallion semen, cryopreservation, cryosurvival, good freezers, single-layer centrifugation, antioxidants, lipid peroxidation, biomarkers, aquaporins, proAKAP4

## Abstract

**Simple Summary:**

Most artificial inseminations in horses currently use cooled semen. It has not been possible to develop a freezing protocol that is suitable for all stallions and all ejaculates. Furthermore, the thawed spermatozoa have a short life, which necessitates depositing the semen in the uterus close to the time of ovulation. Using frozen semen has many potential advantages: there would be greater choice of stallions, the supply of semen is guaranteed, and the amount of antibiotics used would be reduced compared to fresh semen. However, not all ejaculates can be frozen successfully. This review looks at some of the factors that might affect the successful cryopreservation of semen, including stallion nutrition, the frequency of semen collection, the medium that is added to protect the sperm during freezing, the speed of cooling, etc. It would be helpful to identify ejaculates that will freeze well, but as yet, there are no means of identifying them other than carrying out a test freeze.

**Abstract:**

Artificial insemination (AI) is used frequently in the breeding of sport horses, apart from Thoroughbreds. Most AIs are carried out with cooled semen rather than frozen semen because of the difficulties in identifying a protocol that is suitable for freezing most ejaculates and the necessity to inseminate close to ovulation because of the short life of the thawed spermatozoa. More widespread use of frozen semen would improve biosecurity, allow greater choice of stallions, and offer more flexibility when managing deliveries of semen to the stud. It would even decrease the amount of antibiotics used in semen extenders, since the volume of frozen semen is smaller than when cooled semen is inseminated. However, there is considerable variability in the cryosurvival of spermatozoa from different stallions, leading to the classification of stallions as good or bad freezers. Improvements could be made at the level of stallion nutrition, the semen collection regimen, the extender, the removal of seminal plasma, and the cooling protocol, among others. Stallion sperm membranes are highly susceptible to lipid peroxidation, but research on antioxidants has failed to identify an additive that would benefit all stallions. In the future, biomarkers for sperm freezability could be used as an aid in identifying suitable ejaculates for cryopreservation.

## 1. Introduction

The reproductive biotechnology artificial insemination (AI) is widely used for breeding livestock in most parts of the world, and it is also used in horses. Initially, the technique was adopted to reduce disease transmission, since animals can be bred without coming into contact with each other, and transport to different farms for mating is avoided [1]. Subsequently, benefits other than increased biosecurity were identified, including more rapid genetic improvement in livestock than is possible with natural mating [2], as well as safety for both personnel and animals. Success with AI requires, among other factors, a readily available supply of good-quality semen. In cattle breeding, this requirement is met largely by frozen semen.

In contrast, in equine breeding, frozen semen is used less frequently than cooled semen; the use of AI with the latter has increased considerably over the last two decades [3], resulting in a foaling rate of approximately 65% in many countries [4]. The conception rates for mares inseminated with thawed semen are lower than mares inseminated with fresh or cooled semen [5]. Furthermore, it is not possible to freeze all ejaculates successfully; despite decades of research [6], post-thaw sperm quality may be suboptimal, and the timing of the insemination relative to ovulation represents a critical point in the process. Cryopreservation reduces sperm longevity, and there is an increase in oxidative damage from lipid peroxidation of sperm membranes [7]. As a result, the timing of insemination relative to ovulation is more critical with thawed sperm than with cooled semen. In a study by Kuisma et al. [8], the viability of thawed sperm samples was reduced by about 20% during a one-hour incubation. Of course, these negative effects of cryopreservation are not limited to stallion spermatozoa, being seen in many other species too, but since the focus of this review is on freezing equine semen, we confine our observations to this genus.

Although there are other forms of assisted reproduction apart from AI, the latter is by far the most widely used form of ART in equine breeding. Although intracytoplasmic sperm injection (ICSI) is available in specialized laboratories, its use results in only a small proportion of equine pregnancies [9]. In vitro fertilization (IVF) accounts for even fewer pregnancies [9]. Therefore, in this review, we focus on providing sperm for AI, rather than for other forms of ART.

One of the reasons for the low cryosurvival of equine spermatozoa is that, unlike bulls, stallions are chosen as breeding sires based on their performance in competition rather than on the freezability of their ejaculates [10]. There is wide variation in semen quality between individuals [11], which may reflect variations in the antioxidant properties of their seminal plasma, for example.

Since the freezing protocol plays an important role in post-thaw viability, improvements that lead to increased equine sperm cryosurvival would enable more ejaculates from a greater number of stallions to be frozen successfully. Improved post-thaw sperm quality and longevity, resulting in a longer functional life post-insemination, would make the timing of AI relative to ovulation less critical than at present.

Apart from providing a brief summary of the status quo, the purpose of this review is to highlight promising avenues for future research. Progress in existing methodologies is acknowledged, but persisting challenges to be overcome are indicated. The suggested avenues for exploration include factors affecting spermatogenesis and sperm quality pre-freeze, refining cryoprotectant solutions and the cryopreservation process, finding more appropriate biomarkers that can be applied in the field, and fostering interdisciplinary collaboration. It is intended that this review should stimulate the next generation of equine researchers to continue the quest for improved stallion sperm cryosurvival.

## 2. Cryopreservation Method for Stallion Semen

The protocol that is generally used for stallion semen cryopreservation is as follows: semen collection, removal of most of the seminal plasma, resuspension of the sperm pellet in cryoextender, packaging, cooling, and freezing [11,12]. Different rates and methods of cooling and freezing have been advocated, ranging from cooling the sperm suspension in cryomedium for several hours, with or without glycerol (the glycerol-containing medium can be added after the mixture has cooled to 4 °C), to cooling at a controlled rate in a machine. Freezing is performed in liquid nitrogen vapor either by placing the straws at a specific height above the surface of liquid nitrogen or in a freezing machine, where the rate of cooling below 0 °C can be carefully regulated.

## 3. Sperm Quality Pre- and Post-Cryopreservation

There are different interpretations of the expression “sperm quality”; in this review, it is taken to mean an indirect assessment of the potential ability of a sperm sample to achieve fertilization. Thus, factors such as sperm motility, membrane integrity, acrosome integrity, chromatin integrity, and mitochondrial function, are considered to be indicative of future fertilizing potential [13]. Other methods, such as “omics” or molecular assays, require even more advanced instrumentation and expertise. Unfortunately, most stallion semen cryopreservation in the field takes place without recourse to most of these methods of evaluating sperm quality [14]. Instead, sperm quality evaluations are often confined to an assessment of sperm motility (and possibly sperm morphology) in the original ejaculate, and post-thaw motility assessment.

Post-thaw sperm quality in any species is important for success in artificial insemination, but frozen stallion semen tends to present low post-thaw sperm viability, poor motility, and low fertility [11]. Unfortunately, semen quality before freezing is not a guarantee of good quality after cryopreservation [15], but at least it can be used to discard low-quality ejaculates that will likely not freeze successfully. Usually, a post-thaw progressive motility of 35% is considered to be acceptable for AI [11,16].

Some suggestions to improve sperm quality prior to cryopreservation that we discuss in the following paragraphs consider the various stages in the cryopreservation protocol separately. The first of these sections examines factors affecting the semen itself, such as dietary supplements, the season of semen collection, the application of blue light, and the method of semen collection. Once the semen has been collected, various manipulations can affect sperm quality, such as the type of extender used and the removal of seminal plasma and how this is achieved, which is discussed in the second section. The cooling, freezing, and thawing procedures themselves have an effect on post-thaw sperm quality, as does the presence of microorganisms. Finally, some suggestions for promising lines of research are outlined.

## 4. Semen Quality

In this section, various factors affecting the quality of the initial ejaculate are considered, since this is the starting material for the cryopreservation process. Improving sperm quality from the start will likely influence the outcome in the cryopreserved sperm samples.

### 4.1. Dietary Supplements

Some studies have focused on nutraceuticals to improve stallion semen quality and post-thawing viability, adding one or more supplements to stallion diets (Table 1). For a detailed review of nutraceuticals, see Bazzano et al. [17]. The supplements were often polyunsaturated fatty acids and/or antioxidants, or yeast extract. Adding docosahexaenoic acid-enriched nutraceuticals resulted in improved freezability of stallion semen [18], while adding antioxidants (selenium, ascorbic acid, and tocopherol) to the stallions’ diet was considered to improve semen quality [19]. However, another study showed that the combination of linseed oil and antioxidants decreased membrane integrity [20] and had a variable effect on sperm motility depending on the month of investigation, suggesting that there is a delicate balance in the concentrations of antioxidants and polyunsaturated fatty acids required to provide protection. Rodrigues et al. [21] observed that adding polyunsaturated fatty acid to stallion diets improved sperm membrane fluidity and reduced thermal stress, but there was no significant effect on the fresh and cooled semen. Supplementation with plasmolyzed herbal yeast could be useful to improve the antioxidant status of cooled stallion semen but did not affect sperm characteristics [22]. Differing results from various studies may arise from the composition of the control diet; the addition of supplements may not have an effect if the nutritional content of the control diet is adequate. As yet, there are no supplements specifically recommended for improving stallion sperm quality and cryosurvival.

### 4.2. Effect of Season on Sperm Quality

The reproductive activity in equids occurs during a particular period of the year due to the effect of the photoperiod, various hormones (e.g., melatonin, gonadotrophins, steroids, inhibin, prolactin, gonadal steroids, etc.), and endogenous opioids [26]. This seasonal activity is seen in mares; in stallions, sperm production occurs all year round but is reduced during the non-breeding season [27]. Stallions reach full reproductive activity in late spring and summer [28]. Various studies showed that there was a relationship between the time of the year of semen collection and sperm viability after cooling or cryopreservation (Table 2). It is likely that different results may be obtained depending on the study population and methods used for sperm evaluation; even the location of the stallions may play a role.

By collecting semen for freezing every week throughout the year, Magistrini et al. [34] showed that there was little or no difference in post-thaw motility and vitality according to season. Others have reported seasonal influences on sperm progressive motility, viability, and DNA fragmentation [29]. In spring, sperm motility and membrane integrity increased, while sperm DNA fragmentation decreased [35]. However, there were also changes in sperm quality within the breeding season, with lower sperm quality being observed at the beginning and end of the season [36]. Sieme et al. [37] showed that administering exogenous GnRH during the non-breeding season resulted in increased quality of frozen–thawed semen and improved sexual behavior in good freezer stallions, although there was no effect on bad freezer stallions.

For practical reasons, most stallion semen cryopreservation occurs during the non-breeding season, with ejaculates being used for cooled semen doses during the breeding season. However, according to the studies presented in Table 2, post-thaw sperm quality might be enhanced if the semen was frozen during the breeding season, although individual variation might occur.

### 4.3. Application of Blue Light in the Non-Breeding Season

Blue light was reported to have a positive effect on mare reproduction, advancing the breeding season by up to three months [38]. The blue light can be administered from a device attached to the head collar, allowing individual animals to be treated rather than subjecting the whole barn to “light” therapy. There are anecdotal accounts of the effect of blue light on stallion sperm quality; individuals with moderate sperm quality showed an improvement in sperm characteristics, although sperm quality remained unchanged in stallions with good sperm quality. It would be interesting to investigate whether subjecting the stallions to blue light two months prior to semen collection for cryopreservation would improve sperm cryosurvival, at least in some individuals.

### 4.4. Semen Collection Frequency

Sieme et al. [39] compared post-thaw sperm viability from ejaculates collected once daily or twice every other day. The first of the two ejaculates collected every other day had higher values of viable spermatozoa than the ejaculates collected every day [11].

### 4.5. Fractionation of Semen Samples during Collection

In experiments comparing non-fractionated and fractionated semen collection, post-thaw sperm membrane integrity and motility were improved in fractionated samples if the sperm-rich fraction was centrifuged prior to cryopreservation [39]. However, since another study showed that semen collected with an Equidame phantom had better sperm concentration and hygienic quality than semen collected with a conventional Missouri artificial vagina [40], it is not clear whether the increased post-thaw sperm quality in the fractionated samples was due to the separation from seminal plasma or separation from bacteria.

### 4.6. Post-Thaw Sperm Quality

Once the semen has been obtained, post-collection manipulations have a significant effect on post-thaw sperm quality.

#### Removing Seminal Plasma

Seminal plasma is produced from the accessory sex glands, providing metabolic substrates to the spermatozoa, transporting them into the female reproductive tract, and playing a role in sperm maturation [41]. However, removing seminal plasma is important for enhanced sperm quality during storage [11,42]. Protocols for stallion semen cryopreservation remove most of the seminal plasma to increase the sperm concentration, although removing seminal plasma by centrifugation was reported to decrease viability, mitochondrial activity, and acrosomal integrity [43]. Various protocols have been used to remove the seminal plasma for either cooled storage or cryopreservation (Table 3).

In contrast, Al-Essawe et al. [49] tested the effects of adding small amounts of seminal plasma from stallions of known freezability (i.e., “good” or “poor freezers”) to seminal plasma-free samples prepared by Single Layer Centrifugation (SLC) prior to freezing. Although SLC had a beneficial effect on cryosurvival, with increased high mitochondrial membrane potential and decreased hydrogen peroxide production, adding seminal plasma did not have an additional beneficial effect. Adding seminal plasma after thawing was not beneficial [50]. In a heterologous zona binding assay, the sperm samples exposed to seminal plasma did not bind as readily to the zona pellucida of bovine oocytes, suggesting that a longer capacitation time might be required in the presence of seminal plasma [51]. By extrapolation, this effect could lead to an increased length of post-insemination viability, although this speculation requires investigation. Furthermore, Neuhauser et al. [52], studying the effects of adding autologous seminal plasma after thawing on sperm kinematics, concluded that the addition of autologous seminal plasma might be beneficial in assisting sperm motility within the female reproductive tract, particularly for poor freezer stallions. However, the sample size in their study was small, and no fertility trials were carried out.

### 4.7. Sperm Selection

It is generally assumed that there is a reduction of approximately 50% in sperm viability during cryopreservation for any species based on studies conducted with bull spermatozoa [53,54]. Therefore, post-thaw survival is strongly influenced by pre-freeze quality. Selecting motile sperm with good membrane integrity before freezing could be one possibility for maximizing post-thaw sperm quality. Several methods are available for sperm selection (Table 4) which were reviewed previously. Many of these methods result in improved quality in prepared sperm samples, although not all are practical when applied to preparing sperm doses for conventional AI. At present, only colloid centrifugation is used as a sperm selection procedure, while sperm washing (centrifugation and resuspension in fresh extender) with or without a cushion removes most of the seminal plasma without selecting for spermatozoa with desirable characteristics. The theory behind cushion centrifugation is that a high centrifugation force can then be utilized since the spermatozoa are prevented from hitting the bottom of the centrifuge tube by the cushion. Although more spermatozoa can be recovered from the ejaculate with the cushion than without, there may be more damage to the sperm DNA from the high centrifugation force.

Sperm washing is widely used when preparing stallion semen for cryopreservation. However, colloid centrifugation, particularlySLC, has several advantages over sperm washing. The technique is no more complicated than sperm washing—a detailed description is given in Morrell and Nunes [60]—but causes less damage to the spermatozoa and selects robust spermatozoa if a high-density colloid is used. Single layer centrifugation with a low-density colloid to separate the spermatozoa from seminal plasma without any selection for robustness is also possible [61] but has not been tested yet in a cryopreservation protocol.

### 4.8. Semen Extender

Typically, the extenders used for freezing stallion semen contained egg yolk and glycerol. Squires et al. [62] reported that methyl formamide and dimethyl formamide could replace glycerol in the freezing extender for stallion semen, and a commercial extender containing these cryoprotectants was subsequently developed. However, when the egg yolk was replaced by soya bean lecithin, pregnancy rates were reduced, although sperm characteristics were not affected [63]. There were considerable differences between the methods of freezing (controlled versus uncontrolled rate freezing) and the types of extenders that were used, i.e., the glycerol-containing extender, Gent, and BotuCrio, which contains amides [64]. The proportion of spermatozoa with high mitochondrial membrane potential was higher in the Gent extender than in the BotuCrio, and membrane integrity was almost statistically significant in favor of the Gent extender. However, curvilinear velocity and acrosome integrity were higher in the BotuCrio than in the Gent extender.

One of the most important points affecting sperm viability is oxidative stress. Therefore, adding antioxidants to semen extenders is important in improving sperm viability [65,66] (Table 5). Nouri et al. [65] added resveratrol and epigallocatechin-3-gallate to skimmed milk before cryopreserving low-quality semen. Although high and low concentrations of these additives were not beneficial or had no effect, respectively, some concentrations could be identified that were beneficial. However, no fertility data were supplied for this study.

Contreras et al. [66] evaluated the addition of manganese (III) tetrakis (4–69 benzoic acid porphyrin (MnTBAP), N-acetyl cysteine (NAC), and metallo-porphyrin-5,10,15,20-tetrakis(4-sulphonatophenyl) porphyrin iron (III) chloride (FeTPPS) to the freezing medium for stallion spermatozoa, testing the binding capacity of the thawed spermatozoa to bovine oocytes. They observed that MnTBAP was the most effective antioxidant of the three, with higher values for sperm motility, plasma membrane integrity, mitochondrial membrane potential, and the number of spermatozoa binding to the zona pellucida of bovine oocytes, as well as lower lipid disorder.

An alternative to adding antioxidants prior to freezing is to add them after thawing [67,68]. Treulen et al. [68] reported that sperm motility and viability were improved in the presence of MnTBAP, and lipid peroxidation and DNA damage were decreased compared to the controls.

**Table 5:** Composition of cryoextenders.

Original Extender	Modification	Effect	References
INRA 82Frozen storage	Added 2% egg yolk and, after dilution, added 2.5% glycerol	No effect on fertility; improved motility	Vidament et al. [69]
Skim-milk, glucose INRA 96 Frozen storage	Added 2%, 3%, or 4% glycerol	Increased post-thaw motility using 4% glycerol	Scherzer et al. [70]
BotuCrio^®^ Frozen storage	Used soybean lecithin instead of egg yolk	Similar sperm characteristics but reduced fertility rates	Papa et al. [63]
Equiplus Frozen storage	Added 150μM MnTBAP, 1 mM NAC, and 5 μM FeTPPS	Added MnTBAP before freezing improved semen quality	Consuegra et al. [71]
Cooled storage	Added glucose at 0 nM, 67 nM, 147 mM, or 270 mM	Increased total and progressive motility, and curvilinear velocity	Hernandez-Aviles et al. [72]
Freezing without permeating cryoprotectants	Sucrose 100 mM with BSA-1%		Consuegra et al. [71]
Frozen storage	Adding antioxidants	MnTBAP increased post-thaw motility membrane integrity and mitochondrial membrane potential	Contreras et al. [66]
Frozen storage	Adding autologous or heterologous seminal plasma post-thaw	Autologous seminal plasma might improve kinematics	Neuhauser et al. [73]
Frozen storage epididymal sperm samples		Centrifuging in a non-egg yolk extender, followed by freezing in low glycerol medium was beneficial	Neuhauser et al. [74]
Frozen storage	Adding sucrose to BotuCrio	Improved membrane and acrosomal integrity; increase in mitochondrial membrane potential	Moura et al. [75]
Frozen storage	New Caceres extender		Morillo Rodríguez et al. [76]

Note: MnTBAP = manganese (III) tetrakis (4–69 benzoic acid porphyrin).

### 4.9. Rate of Cooling

Slow controlled-rate freezing was found to result in a higher proportion of epididymal spermatozoa being motile after thawing than fast freezing, and the population of spermatozoa with high mitochondrial membrane potential was greater [74]. Furthermore, this result was independent of whether the fast freezing was at a controlled rate or in liquid nitrogen vapor. The viable subpopulation with high mitochondrial membrane potential and low calcium was higher for controlled-rate fast cooling than for controlled-rate slow cooling. Similarly, Heule et al. [77] deduced that slow cooling was better than fast cooling in their experiments.

## 5. Lyophilization

Lyophilization, or freeze-drying, i.e., the process of removing most of the intracellular water from equine spermatozoa, has been attempted, but with limited success. Since spermatozoa are usually immotile after lyophilization, the only possibility of testing their fertilizing capability is through ICSI. Therefore, this topic lies somewhat outside the area covered by this review, which deals with spermatozoa for artificial insemination. However, for completeness, lyophilization has been attempted with equine spermatozoa. Choi et al. [78] described a protocol in which stallion spermatozoa were resuspended in DMEM/F12 medium to which 10% fetal calf serum was added. The samples were placed in a freezer at −20 °C for 24 h before transferring to a precooled freeze-dryer for 48 h with a condensation temperature of −50 °C and vacuum pressure of 150 mTorr. Some blastocysts were obtained after ICSI with the lyophilized sperm samples [78]. The transfer of some of these blastocysts resulted in the birth of two foals, only one of which originated from a lyophilized spermatozoon.

Olaciregui et al. [79] compared the effects of two chelating agents (EGTA and EDTA) on DNA status, evaluated by three separate methods (SCDt, AOT, and DQ). They concluded that there was less DNA damage in the sperm samples lyophilized in EGTA than in EDTA, although different levels of damage were indicated by the different methods of detection. However, the fertilizing ability of the spermatozoa was not tested in their study. 

## 6. Vitrification

Vitrification, a process whereby spermatozoa are frozen ultra-rapidly in a cryoprotectant-free medium to achieve a glass-like state, is an alternative to conventional cryoprotection, using permeating cryoprotectants. Some success was achieved when stallion spermatozoa were vitrified in 0.25 mL straws [71]. However, the protocol requires some optimization. Consuegra et al. [71] obtained better sperm motility by vitrifying stallion sperm samples in spheres instead of straws and used colloid centrifugation to select motile spermatozoa after thawing.

In a recent study [80], Restrepo et al. compared lyophilization, vitrification, and freezing of stallion spermatozoa. They concluded that freeze-drying or freezing caused fewer alterations to spermatozoa than vitrification. Furthermore, freeze-drying resulted in more spermatozoa with a high mitochondrial membrane potential and a higher proportion of non-peroxidized viable spermatozoa than freezing. However, the fertilizing ability of the sperm samples was not tested in their study.

## 7. Bacterial Contamination

One of the challenges in optimizing sperm viability is the presence of microorganisms in semen because they may play a role in harming spermatozoa [81]. Therefore, removing or reducing the bacterial load before cryopreservation may aid sperm cryosurvival. Bacteria are found on the external genitalia and on the mucosa of the distal reproductive tract; most of these represent normal flora [82], while others may be pathogenic [83]. These bacteria pass into the semen during ejaculation; therefore, antibiotics are added to semen extenders to inhibit bacterial growth and reduce the bacterial load [84]. However, antimicrobial agents affected sperm viability [85,86,87], probably because the dead and dying bacteria release reactive oxygen species and intracellular contents that are harmful to viable spermatozoa. Moreover, bacteria may develop resistance [88]. It is questionable whether this nontherapeutic use of antibiotics fits with current recommendations for prudent use of antimicrobial substances [89]. Most of the liquid portion of the inseminate is expelled from the uterus via backflow; thus, the bacteria in the vagina and in the environment are exposed to the antibiotics contained in the insemination dose. The microbiota of the vagina was found to be altered after insemination with stallion semen doses containing antibiotics [90,91], and changes in antimicrobial resistance were detected.

The bacterial load can be removed or reduced by colloid centrifugation [92,93], which could thus lead to a reduction in the use of antimicrobial agents in extenders. As mentioned previously, SLC can be used either to select robust spermatozoa prior to cryopreservation or to separate spermatozoa from the bacteria-containing seminal plasma. In the latter case, a low-density colloid could be used instead of the high-density colloid needed for the selection of robust sperm. When this technique was used to prepare boar semen for AI, there was no detrimental effect on reproductive efficiency in the inseminated sows compared to controls [89].

## 8. Future Directions

There are considerable incentives to be able to use cryopreserved semen more effectively in the equine breeding industry; thus, research into improving sperm cryosurvival is needed. Some of the directions for future research were mentioned already in this review, but using existing techniques in different combinations as a means of improving sperm cryosurvival should not be overlooked. Some possibilities include using blue light to improve semen quality and replacing cushion centrifugation with centrifugation through a low-density colloid as a method of separating spermatozoa from seminal plasma. This substitution may well lead to the generation of fewer ROS, leading to less lipid peroxidation of sperm membranes during cryopreservation. This technique should be tried, for example, with different extenders and different cooling/freezing rate combinations. The potential for supplementing the cryoextender with different antioxidants should be investigated. Many plant-based substances have antioxidant properties and have been tested in semen cryopreservation in several animal species. Although some benefits have been described, they may be species-specific; e.g., rosmarinic acid was considered to enhance boar sperm quality [94] but did not have an effect on bull sperm samples. Combinations of factors should be considered, for example, semen collection routine, antioxidants and freezing rates, or blue light and centrifugation protocols. Above all, one should not forget individual variation [1]; what works for one stallion may not work for another, but this does not negate the usefulness of the method for some stallions. The quest for one universal protocol for cryopreserving stallion semen that will work for all individuals may well be an idealistic endeavor; we can continue to search for it, but, in the meantime, we should focus on doing the best that we can with the material that is available.

### Fertility Biomarkers

Recently, interest in biomarkers has increased, as researchers identify some sperm characteristics that could potentially indicate either freezability or fertility. Such biomarkers could be useful in indicating which ejaculates we should attempt to freeze. Currently, the only method of determining whether an ejaculate is likely to give satisfactory post-thaw results is to perform a test freeze using different cryoextenders. However, not all stud personnel are prepared to go to these lengths; if the ejaculate does not freeze well with their standard protocol, the stallion is not accepted for freezing at that stud. Therefore, a biomarker that could be applied to fresh semen and could serve to indicate whether a stallion’s semen is likely to freeze well could be very useful in determining which ejaculates to take for freezing.

One example of such a biomarker could be aquaporins, proteins that play a role in transporting water and some small molecules in or out of cells. Thus, they may have a crucial role in reducing intracellular water content prior to freezing. They are considered to be involved in a number of functions in the male reproductive system [95,96] and may play a role in protecting stallion spermatozoa against cryodamage [97]. To date, 13 types have been identified [96].

Another potential biomarker is A-kinase anchoring protein, which plays a role in regulating function such as sperm motility and the acrosome reaction [98]. A precursor protein, Pro-AKAP4, has a role in coordinating transduction signals, which are responsible for regulating sperm motility and fertility [99]. Blommaert et al. [100] considered that pro-AKAP4 could indicate the motility of thawed sperm samples, while Carracedo et al. [101] suggested that pro-AKAP4 could be an indicator of the potential motility reserve of the spermatozoa. The concentration of pro-AKAP4 was found to differ depending on the extender used [102]. Thus, pro-AKAP4 could serve as a biomarker for sperm quality and fertility, although it has not been used, as yet, to predict cryosurvival.

Novak et al. [103] identified proteins that were positively or negatively associated with fertility. Some proteins involved in carbohydrate metabolism, and also cysteine-rich secretory protein 3 (CRISP3), were positively related to fertility in their study population, whereas kallikrein-1E2 (KLK2), clusterin, and seminal plasma proteins 1 (SP1) and 2 (SP2) were negatively associated with fertility. However, fertility and sperm cryosurvival are different characteristics. Other investigations identified specific proteins associated with poor total motility, average path velocity, and circular velocity. These comprised secreted phosphoprotein 1, fructose-bisphosphate aldolase, and malate dehydrogenase 1 [104]. In contrast, glutathione peroxidase and triosephosphate isomerase were enriched in samples with higher straight-line velocity values.

In a study involving stallion semen that is or is not tolerant of cooling for cooled storage (i.e., not cryopreservation), several proteins of interest were identified [105]. Annexin A2 was present in semen that does not survive cooling without removal of seminal plasma, whereas peroxiredoxin-6 like protein, transcobalamin-2, and ST3 beta-galactoside alpha-2,3-sialyltransferase 1 were overrepresented in semen that survives cooling well. Since cold storage and cryopreservation are not the same processes, whether or not these proteins can serve as biomarkers of stallion sperm cryosurvival remains to be seen. Clearly, there is still work to be done before proteomics or metabolomics can be applied as biomarkers of stallion sperm freezability [106].

## 9. Conclusions

There are still many factors that might influence stallion sperm cryosurvival—among them are the stallion’s nutrition, semen collection regimen, extender, how seminal plasma is removed, and cooling protocol, among others. Each of these areas could be the subject of future research, providing valuable information that could help cryosurvival. However, it is worth bearing in mind that many semen samples could be frozen and produce acceptable results if laboratory personnel are prepared to test different extenders and protocols. The “one size fits all” concept is not helpful for cryopreserving stallion semen. It would be better to view stallion semen freezing as personalized medicine and to accept that there will be differences between stallions and often between ejaculates, as well.

## Figures and Tables

**Table 1:** Food supplements to improve stallion semen quality.

Supplements (Reference)	Study Period	Semen Type	Effect
Docosahexaenoic acid (Brinsko et al., 2005) [18]	98 days	Fresh Cooled 24 h Cooled 48 h and frozen–thawed	No effect. Increased average path velocity and straightness. Increased total, progressive, and rapid motility.
Linseed oil and antioxidants [20]	84 days	Frozen–thawed	Increased total and progressive motility, curved line velocity, average path velocity, and straight-line velocity in November; decreased total and progressive motility, curved line velocity, average path velocity, and straight-line velocity in February. Membrane integrity was decreased in both November and February.
Polyunsaturated fatty acid [21]	60 days	Frozen–thawed	Increased total and progressive motility.
Plasmolyzed herbal yeast [22]	70 days	Cooled	No significant differences between groups in motility, membrane integrity, or lipid peroxidation. Antioxidant status may be increased.
Vitamin E [23]		Frozen	No effect on post-thaw motility, although motility was improved in cold-stored semen.
Vitamin E, selenium, L-carnitine, and fatty acids [24,25]	60-day crossover trial	Frozen	Higher progressive motility, membrane integrity, acrosomal integrity.

**Table 2:** Summary of seasonal studies on sperm characteristics in cooled and frozen semen.

Type of Semen; Improvement in Sperm Quality Parameters	Winter	Spring	Summer	Autumn	References
Cooled; progressive motility (%)	51	59	45	44	Crespo et al. [29]
Cooled; Progressive motility (%); sperm abnormalities	67.39; 41.52	73.24; 35.29	80.79; 39.18	71.5; 42.63	Waheed et al. [30]
Cooled; intact plasma membrane (%)	79	86	80	75	Crespo et al. [29]
Cooled; %DFI Frozen %DFI	11.3; 12.9	------	25.6; 27.1	------	Mislei et al. [31]
Cooled; viability (%)	58.3	58.1	55.0	58.9	Janett et al. [32]
Frozen; viability (%); motility (%)	65.8, 32.1	------	------	64.2; 39.0	Janett et al. [33]
Frozen; AI doses from one ejaculate	8	9	10	8	Aurich [28]

**Table 3:** Seminal plasma removal and its effects on stallion sperm quality.

Semen Type	Method Used	Effect
Cooled storage for 24 h and 48 h [44]	Centrifugation for 12 min at 400× *g* at room temperature	Increase progressive and total motility in poor coolers in both 24 h and 48 h.
Cushion centrifugation [45]	Compared conical tubes and nipple tubes, centrifuging at 400× or 600× *g*; clear and opaque extenders	Both types of tubes could be used with good results; the opaque extender gave better results in terms of motility than the clear extender.
Cooled storage for 0, 24, 48, 72, and 96 h of storage [43]	Centrifugation	Adverse effect on the acrosomal integrity, mitochondrial activity, and viability.
48 h storage at 4 °C [46]	Centrifugation for 10 min at 600× *g* at room temperature	Reduced velocity.
Fresh and frozen [47]	1—Filtered using a synthetic hydrophilic membrane 2—Centrifugation 600× *g* for 10 min, at room temperature	No difference in sperm quality; more spermatozoa were recovered with the filter.
Frozen [42]	1—Swim-up through a colloid 2—Single-layer centrifugation 3—Sperm washing	Single-layer centrifugation more suitable than other methods.
Frozen [48]	Extender semen centrifuged at 700× *g* for 12 min	Increased membrane integrity, membrane functionality, mitochondrial membrane potential, acrosome integrity, and total and progressive motility.

**Table 4:** Methods used for improving quality of sperm samples or selecting robust spermatozoa.

Methods	References
Swim-up, Percoll gradient, glass wool filtration, Sephadex filtration, Leukosorb filtration, centrifugation	Sieme et al. [55]
Single layer colloid centrifugation	Macias Garcia et al. [56]; Morrell et al. [57]
Discontinuous density gradient centrifugation	Colleoni et al. [58]
Swim-up through a colloid (without centrifugation)	Hidalgo et al. [39]
Modified flotation density gradient centrifugation technique	Umair et al. [59]
Microfluidics	Vigolo et al. [48]

## Data Availability

All data are contained within the article.
